# Physical activity monitors to enhance the daily amount of physical activity in elderly—a protocol for a systematic review and meta-analysis

**DOI:** 10.1186/s13643-018-0733-6

**Published:** 2018-05-02

**Authors:** Rasmus Tolstrup Larsen, Jan Christensen, Carsten Bogh Juhl, Henning Boje Andersen, Henning Langberg

**Affiliations:** 1 CopenRehab, Section of Social Medicine, Department of Public Health, University of Copenhagen, Copenhagen, Denmark; 2 Department of Occupational and Physiotherapy, Copenhagen University Hospital, Copenhagen, Denmark; 3 DTU Management Engineering Inst., Technical University of Denmark, Diplomvej 372 office 226, 2800 Lyngby, Denmark; 4 Research Unit of Musculoskeletal Function and Physiotherapy, Institute of Sports Science and Clinical Biomechanics, Faculty of Health Sciences, University of Southern Denmark, Odense, Denmark; 5 Department of Rehabilitation, Copenhagen University Hospital, Herlev and Gentofte, Denmark

**Keywords:** Elderly, Physical activity, Walking, Physical activity monitors, Feedback, Motivation, Behavioral change, Randomized controlled trials, Systematic review, Meta-analysis

## Abstract

**Background:**

To investigate the use of physical activity monitors (PAMs) for the elderly, the scientific literature should be systematically reviewed and the effect quantified, as the evidence seems inconclusive.

**Methods and design:**

Randomized controlled trials and randomized crossover trials, with participants with a mean age above 65 years, comparing any PAM intervention with other control interventions or no intervention, will be included. This protocol is detailed according to the recommendations of the Cochrane Handbook, and it is reported according to the Preferred Reporting Items for Systematic Reviews and Meta-Analyses Protocols statement.

**Results:**

We will present results from the search in a flow diagram. The results from the analyses will include regular meta-analyses, stratified analyses, and meta-regressions. The results on each outcome of interest will be presented in a summary of findings table.

**Discussion:**

This paper will explore and analyze the heterogeneity of the results and try to identify variables that will enhance the effect of PAMs in elderly. The results will be useful to researchers working with elderly and/or PAMs, health care professionals working with elderly, and relatives together with the elderly themselves.

**Systematic review registration:**

PROSPERO CRD42018083648.

**Electronic supplementary material:**

The online version of this article (10.1186/s13643-018-0733-6) contains supplementary material, which is available to authorized users.

## Background

### Rationale

According to the World Health Organization, elderly who are physically active have lower rates of all-cause mortality and exhibit higher levels of functional health [1]. Even a small change in the daily amount of physical activity may be beneficial on hard outcomes such as all-cause mortality and life expectancy [2]. The American College of Sports Medicine describes walking as the most common type of physical activity among elderly [3] and walking may play a key role in prevention of cardiovascular disease [4].

Physical activity monitors (PAMs) were originally used to quantify the level of physical activity through the amount of steps taken [5] and have been used in research since the 1960s [6]. However, PAMs are also used effectively to motivate and facilitate to an enhanced level of physical activity, as meta-analyses have reported PAM-based interventions to reduce participant weight in weight loss programs [7], increase the level of physical activity [8], and reduce sedentary time significantly [9]. PAMs provide feedback on physical activity [5]. This feedback might facilitate result-driven behavioral change and, hence, increase the level of physical activity [10]. Especially in elderly, PAM-based interventions have been shown to be feasible and effective in enhancing the level of physical activity [11–16]. However, some studies report no significant differences between PAM groups and control groups [17–19]. This could be explained by the devices being difficult and troublesome for elderly to use, and a survey has reported that a third of PAM consumers have stopped using the devices after 6 months [20]. It seems very relevant to investigate the use of PAMs, specifically in elderly as they are expected to behave differently than younger adults. To investigate the above, the scientific literature should be systematically reviewed; the effect quantified and possible factors explaining differences in effect size should be identified.

### Research questions and objective

#### Research questions

1. What is the effect of a PAM-based intervention on physical activity behavior in elderly?
2. What are the potential effects on other outcomes such as changes in body mass index, physical capacity, and health-related quality of life?
3. Which factors explain heterogeneity of the results?

#### Objective

The objective of this systematic review and meta-analysis is to review the literature and estimate the effect on daily level of physical activity, when using PAMs as an intervention, compared to control interventions in participants aged 65 years and over. Furthermore, to investigate if potential physical activity effects can result in changes in secondary outcomes such as body mass index, physical capacity, and health-related quality of life. Lastly, possible factors explaining heterogeneity will be investigated.

## Methods

This protocol is detailed according to the recommendations of the Cochrane Handbook [21], and it is reported according to the Preferred Reporting Items for Systematic Reviews and Meta-Analyses Protocols (PRISMA-P) statement.

### Eligibility criteria

#### Types of studies

Randomized controlled trials (RCTs) and randomized crossover trials will be included.

#### Types of participants

Participants included in eligible studies must have a mean age above 65 years. The participants must be independent walkers with or without walking aids.

#### Types of interventions

For this review, we will include studies comparing any PAM intervention with other control interventions or no intervention. The PAMs may be portable or wearable, electronic or mechanical, and driven by accelerometers, pedometers, or global positioning system (GPS).

#### Types of outcome measures

Studies must report at least one of the primary or secondary outcomes in order to be included.

##### Primary outcome

The primary outcome is changed in daily amount of physical activity. If more than one relevant outcome is reported within a study, the outcome will be extracted or calculated favoring daily number of steps, followed by daily number of meters walked, daily amount of energy expenditure measured as calories, daily metabolic equivalent of task (minutes or hours), and finally, if no objective measure is available, self-reported physical activity.

##### Secondary outcomes

Secondary outcomes include (a) meeting the study-specific recommended level of physical activity, (b) change in time spent sedentary, (c) change in time spent in moderate activity, (d) change in time spent in vigorous activity, (e) change in physical capacity, (f) changes in body mass index, and (g) changes in self-reported outcomes. All secondary outcomes (a to g) are independent from each other and will be extracted and analyzed accordingly.

- Meeting the study-specific recommended level of physical activity, measured objectively by PAMs will be extracted from any study that provides results on this.
- Time spent sedentary will be extracted favoring PAM measured behavior. If no PAM measured behavior exists, self-reported behavior will be used.
- Time spent in moderate activity will be extracted favoring PAM measured behavior. If no PAM measured behavior exists, self-reported behavior will be used.
- Time spent in vigorous activity will be extracted favoring PAM measured behavior. If no PAM measured behavior exists, self-reported behavior will be used.
- Change in physical capacity will be extracted favoring outcomes measured by a cardiopulmonary exercise test and secondly a walking test such as 6-min walking test or similar.
- Changes in body mass index will be extracted from any study that provides results on this.
- Self-reported health-related quality of life will be extracted from any study that provides results on this by using questionnaires. The outcome measurement prioritized in the study will be extracted.

##### Timing of outcome assessment

Data will be extracted at post-intervention and follow-up.

##### Adverse outcomes

All reported adverse events in the eligible studies will be extracted.

### Search methods for identification of studies

#### Electronic searches

Preliminary electronic searches, citation pearl growing, and reference searching have been undertaken to identify relevant references and to screen the papers for relevant search terms. An electronic systematic search for eligible studies in the electronically databases MEDLINE [22], EMBASE [23], SPORTDiscus [24], CINAHL [25], and CENTRAL [26] will be undertaken in March 2018. The search matrix will consist of a combination of relevant keywords and MeSH/Thesaurus terms for (1) population, (2) intervention, and (3) study design. RCTs will be identified using the “The Cochrane highly sensitive search strategies for identifying randomized trials” [27].

No restrictions on language or publication time will be applied. If relevant studies are identified in other language than Danish, English, Swedish, Norwegian, and German, a relevant translator will be contacted. The authors of unobtainable studies or studies with missing data will be contacted.

##### Search strategy for the systematic search

The following search table is used to generate the search strings

**Table:** 

Population	Intervention	Study design
“old adults” [Title/Abstract]	“Step counter” [Title/Abstract]	“randomized controlled trial” [Title/Abstract]
elderly[Title/Abstract]	“physical activity monitor” [Title/Abstract]	“controlled clinical trial” [Title/Abstract]
“Above 60 years” [Title/Abstract]	“step monitor” [Title/Abstract]	“cross-over trial” [Title/Abstract]
Seniors [Title/Abstract]	Pedometer [Title/Abstract]	“cross over trial” [Title/Abstract]
Aged [MeSH]	Fitbit [Title/Abstract]	“randomized” [Title/Abstract]
Aged, 80 and over [MeSH]	“activity monitor” [Title/Abstract]	“clinical trial” [Title/Abstract]
Frail elderly [MeSH]	“nokia go” [Title/Abstract]	“randomly” [Title/Abstract]
*‘older people’.* [Title/Abstract]	“misfit ray” [Title/Abstract]	
*‘older adults’* [Title/Abstract]	“moov now” [Title/Abstract]	
	“accelerometer-based tracker” [Title/Abstract]	
	“xiaomi mi band” [Title/Abstract]	
	“tomtom” [Title/Abstract]	
	“vivoactive” [Title/Abstract]	
	“jawbone” [Title/Abstract]	
	“movement counter” [Title/Abstract]	
	“quantified movement” [Title/Abstract]	
	“fitness tracker” [Title/Abstract]	
	“activity monitoring device” [Title/Abstract] physic* AND activit* AND monitor* [Title/Abstract] PAM[Title/Abstract] AND monitor*[Title/Abstract]	

##### Search string for MEDLINE

(((((((((“randomly” [Title/Abstract]) OR “clinical trial” [Title/Abstract]) OR “randomized” [Title/Abstract]) OR “cross over trial” [Title/Abstract]) OR “cross-over trial” [Title/Abstract]) OR “controlled clinical trial” [Title/Abstract]) OR “randomized controlled trial” [Title/Abstract])) AND ((((((((((((((((((((((PAM[Title/Abstract] AND monitor*[Title/Abstract]))) OR ((physic* AND activit* AND monitor* [Title/Abstract]))) OR “activity monitoring device” [Title/Abstract]) OR “fitness tracker” [Title/Abstract]) OR “quantified movement” [Title/Abstract]) OR “movement counter” [Title/Abstract]) OR “jawbone” [Title/Abstract]) OR “vivoactive” [Title/Abstract]) OR “tomtom” [Title/Abstract]) OR “xiaomi mi band” [Title/Abstract]) OR “accelerometer-based tracker” [Title/Abstract]) OR “moov now” [Title/Abstract]) OR “misfit ray” [Title/Abstract]) OR “nokia go” [Title/Abstract]) OR “activity monitor” [Title/Abstract]) OR Fitbit [Title/Abstract]) OR Pedometer [Title/Abstract]) OR “step monitor” [Title/Abstract]) OR “physical activity monitor” [Title/Abstract]) OR “Step counter” [Title/Abstract])) AND (((((((((“older people” [Title/Abstract]) OR (“older adults”[Title/Abstract]) OR (“old adults”[Title/Abstract]) OR (“residents”[Title/Abstract]) OR elderly[Title/Abstract]) OR “Above 60 years” [Title/Abstract]) OR Seniors [Title/Abstract]) OR Aged [MeSH]) OR frail elderly [MeSH]) OR ((Aged, 80 and over [MeSH])))

##### Search string for CINAHL

(AB “older adults” OR AB “older people” OR “residents” OR “frail elderly” AB “old adults” OR AB elderly OR AB “Above 60 years” OR AB Seniors OR AB Aged OR AB (Aged, 80 and over)) AND (AB “Step counter” OR AB “physical activity monitor” OR AB “step monitor” OR AB Pedometer OR AB Fitbit OR AB “activity monitor” OR AB “nokia go” OR AB “misfit ray” OR AB “moov now” OR AB “accelerometer-based tracker” OR AB “xiaomi mi band” OR AB “tomtom” OR AB “vivoactive” OR AB “jawbone” OR AB “movement counter” OR AB “quantified movement” OR AB “fitness tracker” OR AB “activity monitoring device” OR AB (physic* AND activit* AND monitor*) OR AB (PAM AND monitor*)) AND (AB “randomized controlled trial” OR AB “controlled clinical trial” OR AB “cross-over trial” OR AB “cross over trial” OR AB “randomized” OR AB “clinical trial” OR AB “randomly”)

##### Search string for EMBASE

1. (old adults or elderly or seniors or residents or older adults or older people or frail elderly).ab.
2. ((PAM and monitor*) or (physic* and activit* and monitor*) or “activity monitoring device” or “fitness tracker” or “quantified movement” or “movement counter” or “jawbone” or “vivoactive” or “tomtom” or “xiaomi mi band” or “accelerometer-based tracker” or “moov now” or “misfit ray” or “nokia go” or “activity monitor” or Fitbit or Pedometer or “step monitor” or “physical activity monitor” or “Step counter”).ab.
3. (randomized controlled trial or controlled clinical trial or cross over trial or randomized or clinical trial).ab.
4. aged/
5. controlled clinical trial/ or “randomized controlled trial (topic)”/
6. 1 or 4
7. 3 or 5
8. 2 and 6 and 7

##### Search string for SPORTDiscus

(AB “older adults” OR AB “older people” OR “residents” OR “frail elderly” AB “old adults” OR AB elderly OR AB “Above 60 years” OR AB Seniors OR AB Aged OR AB (Aged, 80 and over)) AND (AB “Step counter” OR AB “physical activity monitor” OR AB “step monitor” OR AB Pedometer OR AB Fitbit OR AB “activity monitor” OR AB “nokia go” OR AB “misfit ray” OR AB “moov now” OR AB “accelerometer-based tracker” OR AB “xiaomi mi band” OR AB “tomtom” OR AB “vivoactive” OR AB “jawbone” OR AB “movement counter” OR AB “quantified movement” OR AB “fitness tracker” OR AB “activity monitoring device” OR AB (physic* AND activit* AND monitor*) OR AB (PAM AND monitor*)) AND (AB “randomized controlled trial” OR AB “controlled clinical trial” OR AB “cross-over trial” OR AB “cross over trial” OR AB “randomized” OR AB “clinical trial” OR AB “randomly”)

##### Search string for CENTRAL

1. Mesh descriptor:[Aged] explode all trees
2. Mesh descriptor:[Frail Elderly] explode all trees
3. “old adults” or elderly or “older adults” or “older people” or “frail elderly” or seniors: ti,ab,kw
4. (PAM and monitor*) or (physic* and activit* and monitor*) or “activity monitoring device” or “fitness tracker” or “quantified movement” or “movement counter” or “jawbone” or “vivoactive” or “tomtom” or “xiaomi mi band” or “accelerometer-based tracker” or “moov now” or “misfit ray” or “nokia go” or “activity monitor” or Fitbit or Pedometer or “step monitor” or “physical activity monitor” or “Step counter”:ti,ab,kw
5. (#1 or #2 or #3) and #4

#### Searching other resources

Searching references of eligible studies and relevant journals by pearl growing will be conducted independently by two reviewers (RTL and JC) in order to include relevant articles not captured by the search strings. The database Clinicatrials.gov will be used to locate ongoing relevant studies.

### Data collection and analysis

#### Data management

The technology platform, *Covidence*, will be used to import citations from the literature searches, screening of title and abstracts, screening of full text, assessing risk of bias in included studies, and extracting the data. The analyses will be conducted in *Stata Statistical Software*, *version 15*.

#### Selection of studies

The selection of studies will be done by merging search results from the databases, removing duplicates, examining the titles, and examining full-text reports according to the inclusion criteria.

Two authors (RTL, JC) will independently screen titles and abstracts and assess eligible articles in full text. Any inconsistencies between authors will be discussed and solved with consultation of a third author (CJ).

#### Data extraction and management

Data on the following items will be extracted from all included studies.

##### Source

Study ID, protocol ID, review author, citation, and contact details

##### Methods

Study design, aim of study, number of arms or groups, funding source, informed consent obtained, and ethical approval

##### Participants

Total number of participants, setting, possible diagnostic criteria, age, sex, country, co-morbidities, education length, and marriage status

##### Interventions

Duration of intervention, specific intervention, and intervention details sufficient for replication

##### Outcomes

All outcomes specified in the “Types of outcome measures” section and specific time points, outcome definitions, and unit of measurement

##### Results

Number of participants allocated to each group, summary data for each intervention, and control groups (as reported) including adverse events

##### Miscellaneous

Funding sources, key conclusions, miscellaneous comments from authors and if correspondence was required

#### Assessment of risk of bias in included studies

Two review authors (RTL and JC) will independently assess the risk of bias in included studies, using the *RoB 2.0 tool* [28]. Disagreement between review authors will be solved by including a third reviewer (CJ). The risk of bias assessment for each study will be presented using a table with judgment and support for judgment.

#### Measures of treatment effect

Treatment effect, measured as continuous data, will be expressed as mean difference with 95% confidence intervals for outcomes measured with the same outcome measurement instrument, or as standardized mean difference with 95% confidence intervals, when different measurement instruments are used in the included studies. Dichotomous outcomes, such as adverse events or meeting the recommended level of physical activity, will be analyzed and expressed as risk ratios with 95% confidence intervals.

#### Unit of analysis issues

##### Studies with multiple treatment groups

If a study has more than one intervention group and both seem relevant for inclusion in the systematic review, the intervention groups will be included as two separate studies and the control group from the study will be separated [29].

#### Assessment of heterogeneity

The heterogeneity of the study results will be examined using Cochrane Q test and quantified with *I*^2^ values and the between study variance tau^2^.

#### Assessment of small study bias

Small study bias will be assessed by calculating an *Egger’s test* score and illustrated with a funnel plot. If small study bias is found, by a positive Egger’s test, a *metatrim* analysis will be conducted and an adjusted effect size will be calculated.

#### Data synthesis

If two or more included studies allows for it, the effect size will be calculated using a random-effects meta-analysis adjusting to Hedges’ *g*. If the included studies need network meta-analysis to be pooled this will be performed, as described in Chapter 16.6.3 of the Cochrane Handbook [30]. An alpha level of 0.05 will be considered statistical significant. If it is not possible to conduct a meta-analysis, we will describe the data narratively specific to each outcome.

#### Subgroup analysis and investigation of heterogeneity

We will explore heterogeneity by conducting sub-group analyses and stratified analyses on the following nominal variables:

- Placement of PAM (ankle worn, wrist worn, hip worn)
- Type of PAM (pedometer versus accelerometer)
- Diagnoses (healthy, cancer patients, pulmonary patients, cardiovascular patients, etc.)
- Feedback frequency (real time, daily, weekly or monthly)

We will explore heterogeneity on continuous data by performing univariate meta-regressions on the following variables:

- Mean age
- Sex distribution
- Number (or percent) of participants with walking aids
- Intervention length
- Mean baseline physical activity

#### Sensitivity analysis

Sensitivity analyses will be performed on all outcomes of interest by stratifying on overall risk of bias, defined by the RoB 2.0 tool [28] (low/some concerns/high). Furthermore, we will perform sensitivity analyses on how the primary outcome has been extracted, by performing meta-analyses with mean differences on (1) change in daily number of steps, (2) change in daily number of meters walked, (3) change in daily amount of energy expenditure measured as calories, (4) change in daily metabolic equivalent of task (minutes or hours), and (5) self-reported physical activity.

#### Summary of findings table

We will create a Summary of findings table with effect sizes on the outcomes earlier presented. Two reviewers (RTL and JC) will independently rate the quality of the evidence using the GRADE approach. Downgrading and upgrading the quality of the evidence will be described in the footnotes in the table. An empty example of a summary of findings table is listed in Additional file 1.

## Discussion

The aim of this systematic review and meta-analysis is to systematically locate, evaluate, summarize, and analyze available evidence regarding the use of PAMs to enhance the level of physical activity in elderly. This paper will explore and analyze the heterogeneity of the results and try to identify variables that will enhance the effect of PAMs in elderly. The discussion will contain a cost-effective evaluation of the effect size of PAMs and the expected prize of the monitors. We anticipate that this review and the results will be useful to researchers working with elderly and/or PAMs, health care professionals working with elderly, and relatives together with the elderly themselves.

### Strength of this systematic review and meta-analysis

We have not identified any systematic reviews investigating the effect of PAM-based interventions in elderly. This paper will be the first systematic review and meta-analysis directly calculating the effect size and exploring heterogeneity of the results. Furthermore, this systematic review and meta-analysis will only include RCTs and randomized crossover trials and hence, the level of evidence is likely to be high.

## Additional file

Additional file 1: **Table S1.** Summary of findings. (DOCX 17 kb)

## Abbreviations

GPS: Global positioning system
PAMs: Physical activity monitors
PRISMA-P: Preferred Reporting Items for Systematic Reviews and Meta-Analyses Protocols
RCTs: Randomized controlled trials
