# Supplementary material for: Physical activity monitors to enhance the daily amount of physical activity in elderly—a protocol for a systematic review and meta-analysis
Source: Syst Rev. 2018 May 2;7:69. doi: 10.1186/s13643-018-0733-6 (PMC5932815; doi:10.1186/s13643-018-0733-6)
Supplement: Supplementary file 1 — Table S1. Summary of findings. (DOCX 17 kb) [file 13643_2018_733_MOESM1_ESM.docx]

**Additional file 1: Table S1 Summary of findings:**

| **Physical Activity Monitors as an Intervention compared to control interventions for increasing the physical activity in the elderly** | | | | | | |
| --- | --- | --- | --- | --- | --- | --- |
| **Patient or population**: increasing the physical activity in the elderly  **Setting**:  **Intervention**: Physical Activity Monitors as an Intervention  **Comparison**: other interventions | | | | | | |
| Outcome № of participants (studies) | Relative effect (95% CI) | **Anticipated absolute effects (95% CI)** | | | Quality | What happens |
|  |  | **Control intervention** | **Physical Activity Monitors as an Intervention** | **Difference** |  |  |
| Change in physical activity:  № of participants: (RCTs) | - |  | - | **0**  (0 to 0) | - |  |
| Risk of meeting the recommended activity level assessed with: Physical activity monitors № of participants: (RCTs) | - |  | - | **0**  (0 to 0) | - |  |
| Change in time spent sedentary: Physical activity monitors.  № of participants: (RCTs) | - |  | - | **0**  (0 to 0) | - |  |
| Change in time spent in moderate activity: Physical activity monitors.  № of participants: (RCTs) | - |  | - | **0**  (0 to 0) | - |  |
| Change in time spent in vigorous activity: Physical activity monitors.  № of participants: (RCTs) | - |  | - | **0**  (0 to 0) | - |  |
| Change in physical capacity:  assessed with: № of participants: (RCTs) | - |  | - | **0**  (0 to 0) | - |  |
| Change in body mass index  assessed with: anthropometrics № of participants: (RCTs) | - |  | - | **0**  (0 to 0) | - |  |
| Change in self-reported HRQoL assessed with: questionnaires № of participants: (RCTs) | - |  | - | **0**  (0 to 0) | - |  |
| Adverse events  № of participants: (RCTs) | - |  | - | **0**  (0 to 0) | - |  |
| ***The risk in the intervention group** (and its 95% confidence interval) is based on the assumed risk in the comparison group and the **relative effect** of the intervention (and its 95% CI).   **CI:** Confidence interval | | | | | | |
| **GRADE Working Group grades of evidence** **High quality:** We are very confident that the true effect lies close to that of the estimate of the effect **Moderate quality:** We are moderately confident in the effect estimate: The true effect is likely to be close to the estimate of the effect, but there is a possibility that it is substantially different **Low quality:** Our confidence in the effect estimate is limited: The true effect may be substantially different from the estimate of the effect **Very low quality:** We have very little confidence in the effect estimate: The true effect is likely to be substantially different from the estimate of effect | | | | | | |
